# Analysis of Clinical, Biopsychosocial Factors and Mmp3 Polymorphism in Women with TMD

**DOI:** 10.3390/diagnostics16142280

**Published:** 2026-07-21

**Authors:** Ian Luna Parente Brasileiro, Francisca Katiana De Oliveira Feitosa Donato, Paulo Henrique Couto Souza, Luciana Reis Azevedo Alanis, Luis Eduardo Almeida, Andrea Duarte Doetzer, Paula Cristina Trevilatto

**Affiliations:** 1 Postgraduate Program in Dentistry, School of Medicine and Life Sciences, Pontifícia Universidade Católica do Paraná (PUCPR), Curitiba 80215-901, PR, Brazil; ian.brasileiro@pucpr.br (I.L.P.B.); francisca.donato@pucpr.edu.br (F.K.D.O.F.D.); couto.s@pucpr.br (P.H.C.S.); l.azevedo@pucpr.br (L.R.A.A.); 2 Oral and Maxillofacial Surgery, School of Dentistry, Marquette University, Milwaukee, WI 53233, USA; luiseduardo.almeida@marquette.edu; 3 Undergraduate Dentistry Course, School of Medicine and Life Sciences, Pontifícia Universidade Católica do Paraná (PUCPR), Curitiba 80215-901, PR, Brazil; duarte.andrea@pucpr.br

**Keywords:** temporomandibular joint, temporomandibular disorders, gene polymorphism, depression, matrix metalloproteinase 3

## Abstract

**Objectives**: Temporomandibular disorder (TMD) is a multifactorial condition that significantly affects patients’ quality of life. This study investigated the association between clinical, biopsychosocial, and genetic factors, including the MMP3 rs679620 polymorphism, and TMD in women from southern Brazil. **Methods**: A cross-sectional case–control study was conducted including 305 women, comprising 138 patients with TMD (disc displacement with or without reduction and arthralgia) and 167 controls without TMD symptoms. TMD was diagnosed according to the Research Diagnostic Criteria for Temporomandibular Disorders (RDC/TMD). Clinical and biopsychosocial characteristics were evaluated, and the MMP3 rs679620 polymorphism was genotyped using real-time polymerase chain reaction (RT-PCR). Univariate and multivariate analyses were performed, with statistical significance established at *p* < 0.05. **Results**: Univariate analysis demonstrated significant associations between TMD and chronic pain (*p* = 0.002), somatic symptoms (*p* < 0.001), and depression (*p* = 0.007). Multivariate analysis identified pain preventing leisure activities, headache, ear ringing (tinnitus), and age as factors significantly associated with the presence of TMD. No significant association was observed between the MMP3 rs679620 polymorphism and TMD in the study population. **Conclusions**: Clinical and biopsychosocial factors were associated with the presence of TMD in this female population, whereas the MMP3 rs679620 polymorphism was not. These findings support the multifactorial nature of TMD and suggest that biopsychosocial factors may play a greater role than the genetic variant investigated in this population. Due to the cross-sectional case–control design, the observed associations should not be interpreted as evidence of causal relationships.

## 1. Introduction

Temporomandibular Disorder (TMD) refers to a set of complex, multifactorial disorders that affect the temporomandibular joint (TMJ), including disc displacement with or without reduction, and is one of the primary non-dental causes of orofacial pain [1,2].

TMD affects individuals’ quality of life, and its pathophysiology is not well established in the literature. Among the most related TMD causes are parafunctional habits, rheumatic diseases, facial trauma, and psychological factors such as emotional stress, depression, and anxiety [3,4,5].

Patients with TMD often report myofascial pain, headache, ear pain, and limited jaw movements [1,6]. Some studies show that approximately 60–70% of the population has at least one symptom associated with TMD, although only about 5% of these individuals require treatment [7]. Additionally, the prevalence of TMD ranges from 3 to 15% in the adult population [8,9,10], with a higher prevalence in women aged 19 to 50 years [11,12], possibly due to hormonal factors [13,14,15].

The mechanisms of TMJ destruction are complex and still poorly understood [16]. An essential aspect in the development and aggravation of TMD is inflammation. Studies demonstrate that an exacerbated inflammatory process is closely linked to the destruction of TMJ anatomical structures [17]. Thus, studies have suggested that, in addition to the aforementioned factors, various genetic polymorphisms [13,15,18,19] may also be involved, each contributing partially to TMJ degeneration [5,20,21,22].

The OPPERA project (Orofacial Pain: Prospective Evaluation and Risk Assessment) is a prospective cohort study that followed 2737 individuals without TMD for an average period of 2.8 years. During this time, 260 people showed the first signs of TMD. In this study, 358 genes were evaluated, including those related to inflammation. The study revealed that there are currently no genetic markers predicting the development of TMD. However, the authors suggested that certain single-nucleotide polymorphisms (SNPs), when combined with specific phenotypes, such as pain, anxiety, depression, and stress, may contribute to TMD [23].

Polymorphisms in the *MMP3* gene, which encodes MMP-3 (stromelysin), have been associated with an increased risk of osteoarthritis, lumbar disc degeneration [24], and Achilles tendon rupture [25]. Increased serum levels of MMP-3 in synovial fluid in knee osteoarthritis have also been observed [26]. In the rs679620 polymorphism of the *MMP3* gene, which is located at 11q22.2 in the exon 2 region, an amino acid substitution occurs, and this change can influence the activation of MMP-3 [26].

Matrix metalloproteinase-3 (MMP-3) plays an essential role in extracellular matrix remodeling by degrading collagen, proteoglycans, laminin, and fibronectin, all of which are important structural components of the temporomandibular joint. Increased MMP-3 activity has been associated with cartilage degradation and inflammatory joint diseases, suggesting that alterations in its activity may contribute to structural changes within the TMJ. The rs679620 polymorphism is a functional missense variant that results in an amino acid substitution, potentially influencing MMP-3 activation and enzymatic activity. Therefore, this polymorphism is a biologically plausible candidate for investigating susceptibility to temporomandibular disorders characterized by disc displacement and arthralgia.

TMD is a public health issue with a significant impact on the quality of life of affected individuals, and an accurate diagnosis in each case is crucial for assigning the best individualized treatment. In this context, the search for genetic markers that identify individuals at higher risk of developing TMD is essential for implementing preventive measures and establishing personalized treatments.

Therefore, the aim of the present study was to investigate the association between the MMP3 gene polymorphism (rs679620) and TMD, as well as clinical and biopsychosocial factors, in a female population from southern Brazil.

## 2. Material and Methods

This case–control study was approved by the Research Ethics Committee of the Pontifical Catholic University of Paraná (PUCPR) and the Evangelical University Hospital of Curitiba (HUEC) under the number 1,533,139. Informed consent was obtained from all patients prior to conducting the study.

Sample selection was conducted using convenience sampling from 2019 to 2021, comprising 305 female patients aged between 17 and 73 years, recruited from the ambulatory care of the HUEC and the Dentistry Clinic of the PUCPR. All patients sought one of the services for dental clinical care or specific treatment due to TMD-related complaints.

The sample was divided into a case group, consisting of 138 women presenting disc displacement with or without reduction and arthralgia (TMD), and a control group, comprising 167 women without any signs or symptoms of TMD.

Patients with chronic use of anti-inflammatories, a history of diabetes, hepatitis, HIV, chemotherapy, malignant disease, autoimmune disease, conditions affecting immunity, pregnancy or lactation, prior TMJ surgery, a previous history of facial trauma, orthognathic surgery, and those with myalgia only (without arthralgia) were not included in the study.

### 2.1. Clinical Analysis

A pre-interview was conducted with all patients to preliminarily assess systemic diseases and to clinically diagnose the presence or absence of TMD. Palpation of the masseter and temporal muscles and of the TMJ was performed to diagnose myalgia and/or arthralgia. The diagnosis of TMD was made using parameters established by the RDC/TMD protocol, a widely used protocol [27]. Palpation, diagnosis, and data analysis were performed by two trained and calibrated professionals (ADD and ILPB), with inter- and intra-examiner Kappa coefficients of 0.95 and 0.96, respectively. Following this analysis, patients answered a questionnaire containing objective questions assessing clinical and psychosocial aspects. The following variables were assessed: (i) age; (ii) oral behaviors; (iii) mandibular functional limitation; (iv) chronic pain; (v) nonspecific physical symptoms (somatic symptoms); (vi) depression; and (vii) anxiety.

For the evaluation of clinical variables, the following tests were used: Oral Behaviors Checklist (OBC), Jaw Functional Limitation Scale (JFLS), Graded Chronic Pain Scale (GCPS), Patient Health Questionnaire 15 (PHQ-15), Patient Health Questionnaire 9 (PHQ-9), Generalized Anxiety Disorder 7 (GAD-7), all of which are part of the RDC/TMD [28].

### 2.2. Genomic DNA Extraction

DNA was collected after patients rinsed their mouths with a 3% glucose solution and then scraped the buccal mucosa with a sterile wooden spatula to obtain buccal cells, in accordance with a protocol established in the literature [29]. Buccal epithelial cells were separated by centrifugation at 2.000 rpm for 10 min. The supernatant was discarded, and the cell pellet was resuspended in 1300 µL of extraction buffer [10 mM Tris-HCl (pH 7.8), 5 mM EDTA, 0.5% SDS]. Next, 10 µL of proteinase K solution (20 mg/mL) was added, and the mixture was kept at 65 °C overnight. DNA was purified by adding 10 M ammonium acetate, precipitated with isopropanol, and resuspended in 50 µL of Tris 10mM (pH 7.6) and EDTA 1mM extraction solution.

### 2.3. Analysis of Functional Candidate Gene Polymorphism

The MMP3 gene marker was selected based on information from the SNPinfo Web Server (https://snpinfo.niehs.nih.gov, accessed on 20 June 2022). The MMP3 gene polymorphism (rs679620) is located on the long arm of chromosome 11, in exon 2, at 11q22.2.

For genotyping, the collected and purified DNA was amplified by real-time PCR using the Applied Biosystems (Waltham, MA, USA) 7500 Real-Time PCR system, and the TaqMan^®^ Genotyping Master Mix from Applied Biosystems was used to identify the studied genotypes.

### 2.4. Statistical Analysis

Statistical analysis was performed using the Statistical Package for Social Sciences (SPSS) version 20.0 for Windows. For the analysis of categorical variables such as oral behaviors, mandibular functional limitation, chronic pain, nonspecific physical symptoms, depression, and anxiety scale, the Pearson chi-square test was applied. The quantitative variable age was categorized into age groups: (i) 17 to 29 years; (ii) 30 to 59 years; and (iii) 60 to 73 years, and was analyzed using the Pearson chi-square test. The genotypic analysis of the tag SNP in the *MMP3* gene was conducted using the Pearson chi-square test. The evaluated data were considered statistically significant when *p* < 0.05.

Multivariate analysis using the backward stepwise method was performed, with variables from the univariate analysis that had *p*-values < 0.20 considered for inclusion. The sample power was calculated based on the variables that remained associated in the multivariate analysis. All variables had a power of 90% or higher.

A limitation of this study is that Hardy–Weinberg equilibrium was not evaluated for the control group. Therefore, the quality and representativeness of the genotypic distribution cannot be formally verified, and the genetic findings should be interpreted with appropriate caution.

## 3. Results

The mean age of the control group (n = 167) was 36.54 ± 15.51 years, and that of the case group (n = 138) was 32.43 ± 12.10 years. Regarding age group, there was no statistically significant difference between groups (*p* = 0.061); however, a lower prevalence of TMD was observed in older age groups (Table 1).

In the case group, 73.2% of women had a score indicating mild parafunctional habits, while in the control group, this value was 80.2%. High levels of parafunctional habits were reported by 26.8% in the case group and 19.8% in the control group. There was no statistically significant difference between the groups (Table 2).

The results on mandibular limitation showed that patients in the case group exhibited severe mandibular functional impairment in 100% of cases, compared with 98.2% in the control group. There was no statistically significant difference between the groups analyzed in this test. Further details are provided in Table 3.

The results related to chronic pain showed that the variables: i. how many days experiencing pain in the last 30 days (*p* = 0.002), ii. pain impacting working (*p* = 0.002), and iii. Pain affecting engagement in leisure activities (*p* = 0.000) showed a statistically significant difference between the case and control groups. Individuals in the case group (14.2%) reported longer periods of pain compared to the control group (4.4%), ranging from 1 to 10 days of pain. Additionally, 22.5% reported that pain prevented them from working, and 36.2% reported that pain prevented them from engaging in leisure activities (Table 4).

Regarding other physical symptoms not directly associated with TMD, the case group reported more such symptoms than the control group, as shown in Table 5 (*p* = 0.000). Experiencing stomach pain (*p* = 0.038), back pain (*p* = 0.003), headache (*p* = 0.000), shortness of breath (*p* = 0.010), ringing in the ears (*p* = 0.000), and dry mouth, blushing, or paleness (*p* = 0.029) were symptoms that showed association with the case group (Table 6).

Table 7 displays the results of the PHQ-9 test, with the depression scale for individuals. Higher levels of depression were more frequent in individuals in the case group compared to the control group (*p* = 0.007).

The results of the GAD-7 showed that both groups had similar moderate levels of anxiety; however, there was a higher prevalence of individuals with high levels of anxiety in the case group (56.5%) compared to the control group (46.1%), although without a statistically significant difference, as shown in Table 8.

Regarding the genetic analysis, there was no statistically significant difference in the genotypic frequency of the rs679620MMP3 polymorphism between the two groups (Table 9).

Twenty samples were excluded from the genetic analysis because genotyping was unsuccessful due to insufficient DNA quality and/or PCR amplification failure.

The multivariate analysis showed that the following variables were associated with TMD: i. pain prevented leisure activities, ii. headache, iii. Ringing in the ears and IV. age (Table 10).

Of the 305 participants initially enrolled in the study, 20 samples were excluded from the genetic analysis because successful genotyping could not be achieved due to insufficient DNA quality and/or PCR amplification failure. Therefore, the genotypic analysis was performed using 285 samples.

## 4. Discussion

This cross-sectional study conducted with women diagnosed with TMD investigated the association between clinical and psychosocial factors and the development of this outcome, and the main results revealed that chronic pain, somatization, and age were associated with the presence of TMD.

It is noteworthy that TMD causes a significant impact on the quality of life of affected individuals, especially under frequent episodes of pain, stress, anxiety, and depression. Studies in different populations have shown that patients with TMD symptoms present compromised sleep and an impact on social activities and emotional balance [30,31,32].

As reported in the current literature, TMD is a condition that predominantly affects female individuals [20,33]. Results from students in Portugal showed that 31.5% of men had some form of TMD, while among women, this value was 68.5% [7]. In Brazil, a study analyzed the medical records of 1000 patients with TMD; 17.7% were men and 82.3% were women [11].

Female patients with TMD tend to present more severe pain conditions when compared to those without TMD. The case group sample consisted only of women with TMD who had arthralgia; patients with only myalgia were not included in the study. For the control group, only women without any signs or symptoms of TMD were included. Thus, it was possible to compare extreme phenotypes and reduce possible biases.

Age has also been reported as a risk factor for TMD. The age range for TMD prevalence among women in the literature is 20–50 years [12,34]. In our study, older patients (60 to 73 years old) had a lower prevalence of TMD (10.9% in the control group vs. 3.6% in the case group). Other studies have shown a relationship between age and TMD, with younger patients tending to present with more symptoms, such as disc displacement and muscle pain, compared with older patients [35,36].

Parafunctional habits, such as bruxism, are often associated with TMD, being considered one of the main causes and aggravations of symptoms related to this condition [6], and have already been especially evidenced in adolescents [4,37]. As reported in the literature [38], all patients in this study exhibited some degree of parafunction, whether mild or severe, in both groups. There was a slight tendency for TMD patients to exhibit greater parafunction, although no statistically significant difference was observed between the groups. Patients with TMD commonly report pain, which may be associated with increased stress levels and parafunctional behaviors.

The JFLS test, which assesses mandibular functional limitation, showed similar results for the case and the control groups. These results suggest that mandibular limitation was not significantly associated with the presence of TMD in the study sample. Nguyen et al., who evaluated bone changes in the TMJ of elderly individuals with TMD using the same assessment instrument, found similar results [39]. Another study, which evaluated the masticatory function of patients with and without TMD using electromyography of the masseter muscle, concluded that although food consistency influenced masticatory cycles, the behavior of the masticatory muscles in both groups was similar [40]. Additionally, it is common for TMD patients to present with muscle pain, which can impair mandibular function; thus, these patients tend to make dietary changes [41], consuming softer foods, avoiding foods that exacerbate pain, and minimizing discomfort during chewing.

Chronic pain also has been associated with TMJ disorders. In 2019, studies conducted by Canales et al. [42] showed that 15.2% of the participants reported high-intensity pain levels, with persistent pain more prevalent in the case group. Our results showed that the case group had longer periods of pain than the control group over 30 consecutive days. It is important to note that the pain reported by the patients in this study was not necessarily in the TMJ region, since the control group was composed only of patients without TMJ pain. Psychosocial impairment has also been associated with TMD [42,43], affecting the quality of life of these individuals, which can impair socialization and even feeding. In this study, 22.5% of TMD patients reported debilitating pain while performing work activities, and 36.2% reported being unable to perform leisure activities due to pain.

Regarding nonspecific (somatic) physical symptoms, some studies show that patients with TMD have higher levels of somatization than those without TMD [44,45]. In this study, 19.6% of individuals in the case group exhibited high levels of somatization, consistent with findings reported in other studies [44,45]. The associated symptoms were: stomach pain, back pain, headache, difficulty breathing, ringing in the ears, and a sensation of dry mouth, flushing, or paleness. Those symptoms, especially when chronic, end up affecting patients emotionally. This process may increase stress levels, which can lead to parafunction. As a result, those deleterious effects on the TMJ contribute to the onset or worsening of symptoms related to TMD.

Depression is associated with a significant portion of TMD cases [9,39,46]. Nguyen et al. evaluated the relationship between depression and anxiety with bone changes in the TMJ, and 12.3% of the participants reported a moderate degree of depression [39]. Other studies have reported rates of 30.4%, 19%, and 20.7% [8,9,47]. In our study, 51.4% of patients with TMD presented severe depression, while in the control group, 35.9% reported the same index. This discrepancy in results between studies probably occurs due to the clinical moment in which the data were collected; a great part of the sample was collected during and after the COVID-19 pandemic. Also, the self-perception of depression can vary greatly among individuals.

In addition to depression, anxiety is also a commonly reported factor associated with the presence of TMD. This condition appears to be associated not only with the onset of TMD symptoms but also with their maintenance and worsening [4,6]. Studies suggest that patients affected by depression and anxiety present more symptoms of TMD [9]. It is believed that anxiety, along with stress, leads to muscle hyperactivity and TMJ dysfunctions [4]. Our results showed no association of anxiety with TMD, corroborating with other studies [48,49].

After multivariate analysis, the following variables remained associated with TMD: i. pain prevented leisure, ii. headache, iii. ringing in the ears, and iv. age. Higher levels of chronic TMJ pain and depression have been associated with ear ringing in patients affected by TMD [50]. Tinnitus is defined as sounds perceived by individuals, even in the absence of sound stimuli, which can be triggered by the activity of the muscles of the TMJ [51]. Regarding age, younger patients tend to develop more TMD-related signs and symptoms requiring treatment, whereas in older patients, it is more common to present with more clinical signs but fewer symptoms requiring treatment [52].

This study has several limitations. First, participants were recruited using convenience sampling from university dental clinics and hospital outpatient services, which may limit the generalizability of the findings to the broader population. Second, data collection occurred between 2019 and 2021, overlapping with the COVID-19 pandemic. The psychological impact of the pandemic may have influenced participants’ anxiety and depression scores independently of TMD status, representing a potential confounding factor. Additionally, anxiety and depression were assessed using self-reported questionnaires, which reflect participants’ perceptions at the time of evaluation and may not fully represent long-term psychological status. Finally, because this was a cross-sectional case–control study, the observed relationships should be interpreted as associations and do not establish causal relationships between the evaluated variables and TMD.

The etiology of these conditions is complex and multifactorial, involving genetic and environmental factors [53,54]. The lack of a genetic association identified in this study highlights the need for further research to explore other genes and molecular pathways, as well as interactions between genetic and environmental factors involved in the etiology of TMJ disorders [55].

## 5. Conclusions

The findings of the univariate analysis showed that chronic pain, physical symptoms, and depression were associated with the presence of TMD. After the multivariate analysis, pain-preventing leisure, headache, ear ringing, and age were associated with the presence of TMD. However, no association was observed between the MMP3 gene polymorphism (rs679620) and TMD in the study sample.

## Figures and Tables

**Table 1:** Age by group.

Sample Size (n = 305)	Control n (%)	Case n (%)	*p* Value *
17 to 29 years	80 (48.5)	72 (52.6)	0.061
30 to 59 years	67 (40.6)	60 (43.8)	0.061
60 to 73 years	18 (10.9)	5 (3.6)	0.061

* Pearson’s Chi-square test.

**Table 2:** Oral Behaviors Checklist (OBC) test result.

Sample Size (n = 305)	Control n (%)	Case n (%)	*p* Value *
Mild	134 (80.2)	101 (73.2)	0.145
High	33 (19.8)	37 (26.8)	0.145

* Pearson’s Chi-square test.

**Table 3:** Jaw Functional Limitation Scale (JFLS-20) test result.

General Data (n = 305)	Control n (%)	Case n (%)	*p* Value *
Moderada-Grave	3 (1.8)	0 (0.0)	0.114
Grave	164 (98.2)	138 (100.0)	0.114

* Pearson’s Chi-square test.

**Table 4:** Graded Chronic Pain Scale (GCPS) test result.

General Data (n = 305)	Control n (%)	Case n (%)	*p* Value *
How many days did you have pain in the last 30 days?			0.002
Zero	149 (94.3)	108 (80.6)	
1 to 10 days	7 (4.4)	19 (14.2)	
11 to 20 days	2 (1.3)	3 (2.2)	
21 to 30 days	0 (0.0)	4 (3.0)	
Did your pain prevent you from working?			0.002
Yes	16 (9.7)	31 (22.5)	
No	149 (90.3)	107 (77.5)	
Did your pain prevent you from engaging in any leisure activity?			0.000
Yes	16 (9.7)	50 (36.2)	
No	149 (90.3)	88 (63.8)	

* Pearson’s Chi-square test.

**Table 5:** Patient Health Questionnaire 15 (PHQ-15) test result.

General Data (n = 305)	Control n (%)	Case n (%)	*p* Value *
Normal	38 (22.8)	9 (6.5)	0.000
Low	67 (40.1)	50 (36.2)	0.000
Medium	47 (28.1)	52 (37.7)	0.000
High	15 (9.0)	27 (19.6)	0.000

* Pearson’s Chi-square test.

**Table 6:** Patient Health Questionnaire 15 (PHQ-15) variables test results.

General Data (n = 305)	Control n (%)	Case n (%)	*p* Value *
Stomach pain			0.038
Never	99 (59.3)	62 (44.9)	
Sometimes	55 (32.9)	64 (46.4)	
Always	13 (7.8)	12 (8.7)	
Back pain			0.003
Never	50 (29.9)	20 (14.5)	
Sometimes	84 (50.3)	76 (55.1)	
Always	33 (19.8)	42 (30.4)	
Joint pain			0.525
Never	69 (41.6)	48 (35.3)	
Sometimes	73 (44.0)	65 (47.8)	
Always	24 (14.5)	23 (16.9)	
Menstrual cramps			0.207
Never	71 (43.0)	48 (34.8)	
Sometimes	69 (41.8)	60 (43.5)	
Always	25 (15.2)	30 (21.7)	
Headache			0.000
Never	38 (23.0)	8 (5.8)	
Sometimes	5 (3.0)	1 (0.7)	
Always	122 (73.9)	128 (93.4)	
Chest pain			0.117
Never	132 (80.5)	97 (70.3)	
Sometimes	30 (18.3)	38 (27.5)	
Always	2 (1.2)	3 (2.2)	
Dizziness			0.068
Never	98 (59.0)	64 (46.4)	
Sometimes	63 (38.0)	66 (47.8)	
Always	5 (3.0)	8 (5.8)	
Fainting spells			0.810
Never	160 (96.4)	130 (94.9)	
Sometimes	1 (0.6)	1 (0.7)	
Always	5 (3.0)	6 (4.4)	
Feeling your heart pound or race			0.617
Never	96 (57.8)	73 (52.9)	
Sometimes	63 (38.0)	60 (43.5)	
Always	7 (4.2)	5 (3.6)	
Shortness of breath			0.010
Never	123 (74.5)	82 (59.4)	
Sometimes	33 (20.0)	49 (35.5)	
Always	9 (5.5)	7 (5.1)	
Pain or problems during sexual intercourse			0.088
Never	146 (88.0)	111 (80.4)	
Sometimes	16 (9.6)	25 (18.1)	
Always	4 (2.4)	2 (1.4)	
Constipation, Loose bowels or diarrhea			0.118
Never	98 (59.0)	76 (55.1)	
Sometimes	56 (33.7)	58 (42.0)	
Always	12 (7.2)	4 (2.9)	
Nausea, gas or indigestion			0.858
Never	82 (49.4)	64 (46.4)	
Sometimes	73 (44.0)	65 (47.1)	
Always	11 (6.6)	9 (6.5)	
Ringing in the ears			0.000
Never	129 (78.2)	63 (45.7)	
Sometimes	31 (18.8)	50 (36.2)	
Always	5 (3.0)	25 (18.1)	
Hot or cold flashes, blurred vision, cramps			0.086
Never	82 (50.0)	52 (37.7)	
Sometimes	72 (43.9)	73 (52.9)	
Always	10 (6.1)	13 (9.4)	
Dry mouth, blushing or paleness			0.029
Never	95 (57.6)	68 (49.3)	
Sometimes	65 (39.4)	56 (40.6)	
Always	5 (3.0)	14 (10.1)	

* Pearson’s Chi-square test.

**Table 7:** Patient Health Questionnaire 9 (PHQ-9) test result.

General Data (n = 305)	Control n (%)	Case n (%)	*p* Value *
Normal	3 (1.8)	0 (0.0)	0.007
Mild	21 (12.6)	7 (5.1)	0.007
Moderate	83 (49.7)	60 (43.5)	0.007
Severe	60 (35.9)	71 (51.4)	0.007

* Pearson’s Chi-square test.

**Table 8:** Generalized Anxiety Disorder 7 (GAD7) test result.

General Data (n = 305)	Control n (%)	Case n (%)	*p* Value *
Normal	13 (7.8)	6 (4.3)	0.149
Mild	18 (10.8)	8 (5.8)	0.149
Moderate	59 (35.3)	46 (33.3)	0.149
Severe	77 (46.1)	78 (56.5)	0.149

* Pearson’s Chi-square test.

**Table 9:** Genotypic analysis of rs679620 in the MMP3 gene.

Gene	SNP	Groups (n = 285)α	Homozygous TT n (%)	Heterozygous TA n (%)	Homozygous AA n (%)	*p* Value *
MMP3	rs679620	Control	27(17.4)	97(62.6)	31(20.0)	0.631
MMP3	rs679620	Case	28(21.5)	75(57.7)	27(20.8)	0.631

* Pearson’s Chi-square test.

**Table 10:** Multivariate analysis results.

Variable	*p* Value	Odds Ratio	CI
Pain prevented leisure (1)—No	0.000	4.80	2.27–10.1
Headache	0.000	-	-
Headache (1)—No	0.368	0.32	0.02–3.72
Headache (2)—Sometimes	0.000	5.02	2.05–12.32
Ringing in the ears	0.000	-	-
Ringing in the ears (1)—No	0.001	2.69	1.49–4.85
Ringing in the ears (2)—Sometimes	0.001	6.82	2.26–20.5
Age	0.045	-	-
Age (1)—17 to 29 years	0.062	3.97	0.93–16.97
Age (2)—30 to 59 years	0.294	2.15	0.51–8.97

## Data Availability

Upon reasonable request, the authors will share the data from the questionnaires used. Ethical permission or patient consent does not permit sharing the complete data. The datasets analyzed during the current study are available in the NCBI dbSNP repository, [https://www.ncbi.nlm.nih.gov/snp/rs679620] (accessed on 25 May 2026).

## Abbreviations

COVID	Coronavirus Disease
DNA	Deoxyribonucleic Acid
GAD-7	Generalized Anxiety Disorder 7
GCPS	Graded Chronic Pain Scale
HUEC	Evangelical University Hospital of Curitiba
JFLS	Jaw Functional Limitation Scale
MMP3	Matrix Metalloproteinase 3
OBC	Oral Behaviors Checklist
OPPERA	Orofacial Pain: Prospective Evaluation and Risk Assessment
RT-PCR	Real-Time Polymerase Chain Reaction
PHQ-9	Patient Health Questionnaire 9
PHQ-15	Patient Health Questionnaire 15
PUCPR	Pontifical Catholic University of Paraná
RDC/TMD	Research Diagnostic Criteria for Temporomandibular Disorders
SNPs	Single-Nucleotide Polymorphisms
TMD	Temporomandibular Disorder
TMJ	Temporomandibular Joint
